# Improved Description of Environment and Vibronic Effects with Electrostatically Embedded ML Potentials

**DOI:** 10.1021/acs.jpclett.4c02949

**Published:** 2025-01-13

**Authors:** Kirill Zinovjev, Carles Curutchet

**Affiliations:** † Departamento de Química Física, Universidad de Valencia, 46100 Burjassot, Spain; ‡ Departament de Farmàcia i Tecnologia Farmacèutica, i Fisicoquímica, Facultat de Farmàcia i Ciències de l’Alimentació, 16724 Universitat de Barcelona (UB), 08028 Barcelona, Spain; § Institut de Química Teòrica i Computacional (IQTCUB), 16724 Universitat de Barcelona (UB), 08028 Barcelona, Spain

## Abstract

Incorporation of environment and vibronic effects in simulations of optical spectra and excited state dynamics is commonly done by combining molecular dynamics with excited state calculations, which allows to estimate the spectral density describing the frequency-dependent system-bath coupling strength. The need for efficient sampling, however, usually leads to the adoption of classical force fields despite well-known inaccuracies due to the mismatch with the excited state method. Here, we present a multiscale strategy that overcomes this limitation by combining EMLE simulations based on electrostatically embedded ML potentials with the QM/MMPol polarizable embedding model to compute the excited states and spectral density of 3-methyl-indole, the chromophoric moiety of tryptophan that mediates a variety of important biological functions, in the gas phase, in water solution, and in the human serum albumin protein. Our protocol provides highly accurate results that faithfully reproduce their *ab initio* QM/MM counterparts, thus paving the way for accurate investigations on the interrelation between the time scales of biological motion and the photophysics of tryptophan and other biosystems.

## Graphical abstract

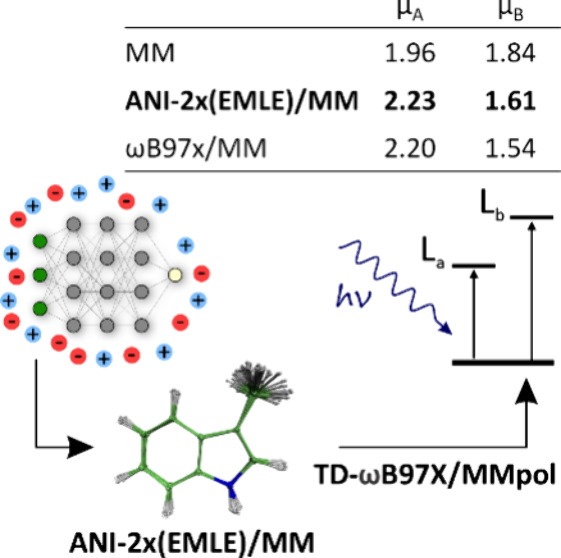

Simulations of optical spectra and excited state dynamics observed in linear and nonlinear spectroscopies depend on the coupling between excited states and internal and environmental vibrations.^1,2^ This is particularly interesting in biological systems, where heterogeneities in the environment can introduce subtle differences in the photoinduced behavior of a given chromophore. The observation of quantum beatings in the 2D electronic spectra of photosynthetic complexes, for example, has motivated extensive studies on the role of exciton-bath interactions and quantum coherence on the optimization of photosynthetic electronic energy transfer.^3–6^

Vibronic effects determine the homogeneous broadening of spectral lineshapes, whereas environment effects lead to inhomogeneous broadening and solvatochromic shifts. Incorporation of these effects in theoretical simulations is commonly done combining molecular dynamics (MD) with subsequent excited state calculations on a representative number of snapshots.^7–10^ Advanced multiscale excited state methods like polarizable embedding now allow highly accurate excited state calculations in condensed phases.^9,11^ Sampling the configurational space, however, still represents a challenge, especially when dealing with large biosystems characterized by motions in multiple time scales.^12^ In such cases, the need for efficient sampling leads to the common adoption of classical force fields, despite well-known inaccuracies in the resulting excited state properties due to the geometry mismatch between the classical potential and the excited state method. A remedy for this problem relies on collecting a number of snapshots from the trajectory and performing multiple quantum/molecular mechanical (QM/MM) geometry optimizations of the chromophores.^1,13–15^ This allows an accurate description of environment effects, and vibronic effects and the Franck–Condon spectrum can later be obtained by computing the normal modes of the ground and excited states, or only the ground state in vertical gradient methods, assuming a displaced harmonic oscillator model.^1,16^

The main limitation of vibronic models based on normal-mode analysis is that the environment and vibronic effects are not treated consistently. Instead of performing multiple QM/MM optimizations along classical trajectories, it is thus more attractive to directly perform QM/MM MD simulations, avoiding the use of classical potentials. Fourier transform of the classical autocorrelation function of energy gap fluctuations then allows an estimation of the spectral density (SD), *J*(ω), a function that encapsulates the information on the frequency-dependent coupling between nuclear degrees of freedom and the excitation.^12,17–21^ Whereas deriving spectral densities from classical trajectories have been shown to lead to considerable errors, recent studies have shown that highly accurate SDs can be derived from Born–Oppenheimer MD QM/MM simulations based on semiempirical or density functional theory (DFT) potentials.^15,22–25^ By using a quantum mechanical (QM) method for the photoactive region, the sampled conformations explore a potential energy surface (PES) more consistent with the QM excited state method of choice, resulting in more reliable predictions of excitation energies, spectral densities, and other properties like transition dipole moments. However, the cost of high-level QM/MM potentials is still too high for routine simulations, especially in multichromophoric systems like photosynthetic complexes, limiting the choice of QM Hamiltonian often to cheap semiempirical methods.

A possible solution to the high cost of QM/MM simulations is to employ a machine learning (ML) potential. The promise of ML potentials (MLPs) is to provide energies and geometries with quality comparable to the reference QM method used for model training while at significantly lower computational cost.^26–28^ Nevertheless, precise MLPs are still computationally much more expensive than molecular mechanics (MM) force fields. This is particularly the case for biomolecular simulations, where the size of the system and large variety of atomic environments make the description of the entire system with an MLP impractical. Moreover, MLPs are not designed to accurately handle long-range interactions, which are key in biosystems. A convenient strategy thus relies on developing hybrid ML/MM approaches that, similar to QM/MM, allow employment of ML potentials only for the region of the system where a precise description is necessary, while treating the rest with a cheap MM force field.^29^ Among these approaches is the recently introduced electrostatic machine learning embedding (EMLE) scheme.^30,31^ EMLE has three key advantages. First, it explicitly treats the polarization of the ML part using a model based on atomic polarizabilities.^32,33^ Second, it treats the impact of the MM environment as a correction term to the *in vacuo* energy of the ML part. This allows one to employ ML potentials that were trained to predict gas phase energies (as most existing ML potentials are) without considering the presence of the MM environment. Third, it is designed to be used as a drop-in replacement for a QM engine in standard electrostatic embedding. Therefore, it can be directly employed in existing QM/MM codes without any modifications. As a result, EMLE allows ML/MM simulations to be run with existing gas phase ML potentials and standard QM/MM MD packages, without any code changes.

Here, we combine EMLE simulations with the QM/MMPol polarizable embedding model^11,34^ to compute the excited states and the spectral density of a key biological chromophore, 3-methyl-indole (3MI), in aqueous solution and in the protein human serum albumin (HSA), following the protocol outlined in Figure 1. 3MI represents the chromophoric moiety of tryptophan, which mediate a variety of important biological functions. After UV light absorption, tryptophan shows a strong fluorescence that is highly sensitive to the surrounding environment, which explains its wide use as a probe to inspect and monitor protein structures and biological processes.^35,36^ Accurately understanding how tryptophan photochemistry is tuned by different biological microenvironments is thus of interest for a variety of techniques. We thus generated structures of 3MI solvated in water and embedded in the HSA protein at the ANI-2x/MM level of theory for subsequent calculation of excitation energies and transition moments with QM/MMPol.^34^ ANI-2x is a generic gas phase ML potential trained on ωB97*X*/6-31G­(d) energies of small molecules (including H, C, N, O, S, F, Cl).^37^ To separately estimate the importance of a good *in vacuo* potential and of a correct treatment of embedding in different environments, three separate sets of simulations were performed. First, we compared the performance of ANI-2x to classical MM and *ab initio* potentials by propagating the MD trajectories of 3MI *in vacuo* using the Amber ff14SB^38^ force field and the ωB97X functional with the 6-31G­(d) basis set, respectively.^39^ In MM simulations, thus, 3MI was described adopting the bonded and Lennard-Jones parameters corresponding to the side chain of Trp in ff14SB, whereas RESP charges were derived at the HF/6-31G­(d) level of theory on a B3LYP/cc-pVTZ optimized geometry. In this case, the simulation system contained only 3MI, so no solvent was present and no embedding scheme was employed. Five replicas of 20 ps were obtained with each Hamiltonian, taking snapshots every 5 fs. The second and third sets of simulations followed the same protocol, but the system contained solvated 3MI or HSA bound to (S)-flurbiprofen. In all cases, MM atoms within 12 Å of 3MI were considered in QM or ML calculations. For the simulations of HSA, we started from an equilibrated structure taken from previous work.^40^ Water was described by the TIP3P model,^41^ whereas for the protein and flurbiprofen we adopted Amber ff12SB parameters. In HSA simulations, the 3MI side chain of Trp214 was chosen as the chromophore described using QM, MM, or ML potentials, and QM/MM and ML/MM boundaries were treated using the link atom scheme.

**Figure 1:**
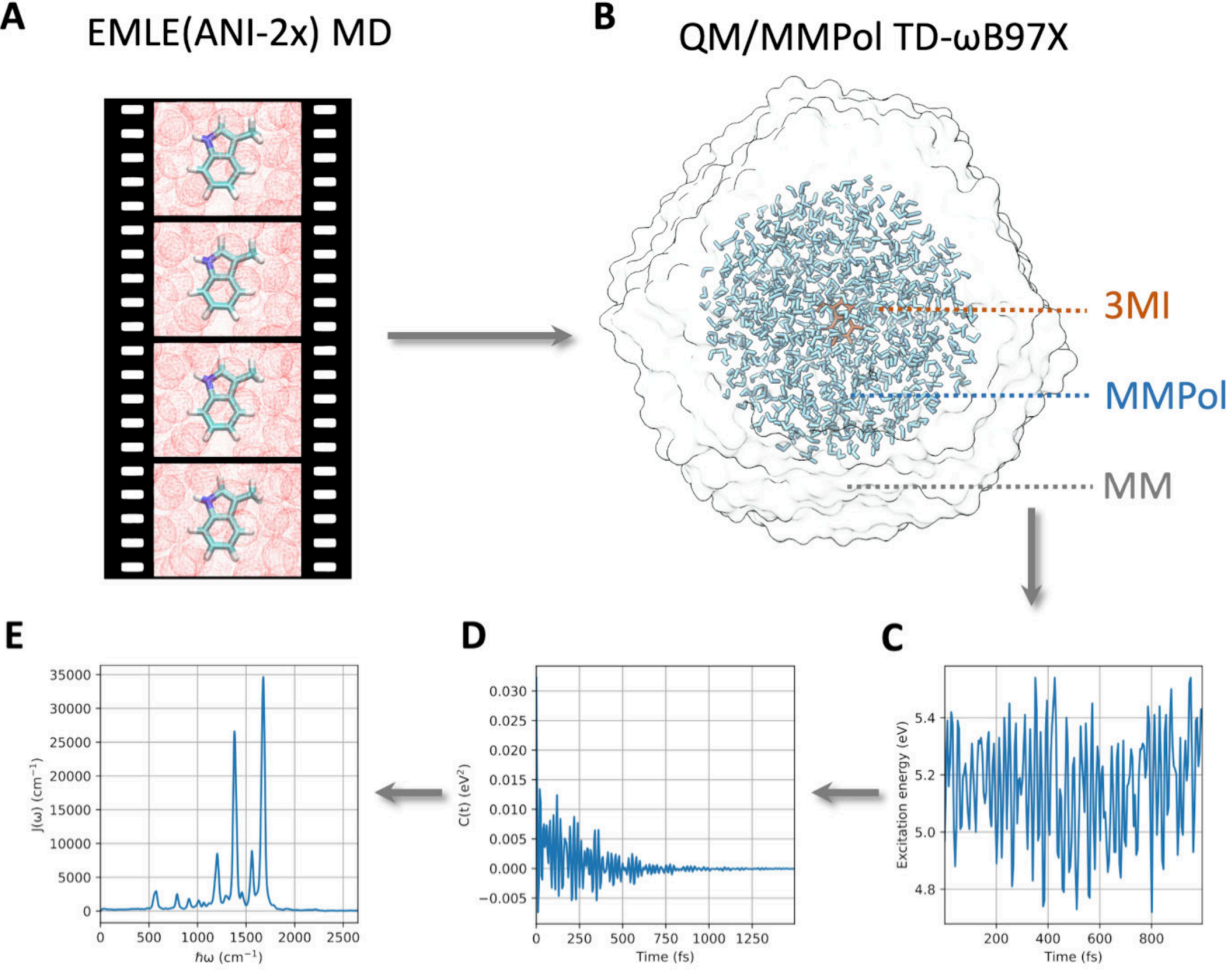
Protocol for the simulation of the spectral density of vibronic coupling of 3-methyl-indole based on EMLE MD simulations and QM/MMPol excited state calculations. (A) ANI-2x­(EMLE) MD simulations are performed to sample the vibrations of the chromophore embedded in the MM environment. (B) The trajectory is postprocessed performing TD-ωB97X QM/MMPol excited state calculations every 5 fs. (C) Fluctuations of excitation energies are computed along the MD trajectories. (D) Autocorrelation function of excitation energy fluctuations along the MD trajectories. (E) Spectral densities of vibronic coupling are derived from the Fourier transform of the autocorrelation function of excitation energy fluctuations.

All simulations were performed using the sander MD code from the AmberTools23 package.^42^ Reference QM/MM calculations were performed using the ORCA package.^43^ In the case of simulations involving the ANI-2x potential, sander was coupled to the emle-engine^31^ code. emle-engine implements EMLE embedding and provides an interface to the torchani^44^ package implementing the ANI-2x potential.^37^ In all cases, the snapshots were used to calculate excitation energies (Δ*E*) and transition dipole moments (μ^T^) at the ωB97X/6-31G­(d) level of theory using the time-dependent density functional theory (TD-DFT) implementation of QM/MMPol in the development version of Gaussian.^45^ For comparison, we also calculated the excited states using the B3LYP hybrid functional. The MMPol environment was described using the Amber pol12 AL polarizable force field, with water charges taken from previous work^46^ and polarization-consistent ESP charges for flurbiprofen derived at the B3LYP/6-31G­(d) level of theory using the Polchat tool.^47^ We used MMPol and MM cutoff radius values equal to 15 and 30 Å, respectively. 3MI has two nearly degenerate π–π* excited states that dictate low-energy absorption and fluorescence, termed L_a_ and L_b_ according to the Platt nomenclature.^48^ These states were carefully identified in each snapshot from the two lowest-lying excited states based on the orientation of the transition dipoles. We used as references the position vector from atom NE1 to CE3 to assign L_a_, and the vector CG to CZ2 to assign L_b_, where the atom names correspond to the Trp definition in Amber force fields. In our simulations, the orientations of the L_a_ and L_b_ states deviated an average of ∼10–20° and ∼30–45° from the references, respectively, indicating that state mixing was small. In Figure S5 from the Supporting Information, we show examples of the L_a_/L_b_ energies in trajectories obtained in different environments.

Spectral densities of vibronic coupling^18,49^ were then computed from the Fourier transform of the autocorrelation function of excitation energy fluctuations along MD trajectories:

$$J\left(\omega \right)=\frac{\beta \omega }{\pi }\int_{0}^{\infty }C^{cl}\left(t\right)\;\cos\left(\omega t\right)\;dt$$ 1

where β = 1/(*k*_B_ *T*) and *C* ^cl^(*t*) is the classical autocorrelation function:

$$C_{i}^{cl}\left(t_{j}\right)=\frac{1}{N-j}\overset{N-j}{\underset{k=1}{\sum }}\Delta E_{i}\left(t_{j}+t_{k}\right)\Delta E_{i}\left(t_{k}\right)$$ 2

The autocorrelation function for the L_a_ and L_b_ states of 3MI was found to decay within 1 ps, so it was averaged over multiple windows of 4 ps following the protocol reported previously.^22^

For the sake of comparison, we also computed the SD of 3MI under a vacuum using the vertical gradient (VG) method from a normal-mode analysis of the ground state and TD-DFT calculations performed at the ωB97X/6-31G­(d) level of theory. The resulting SDs were broadened using a Lorentzian line shape with HWHM values of 15 and 35 cm^–1^.^2,46^

In the case of the solvated system, different ML embedding options were tested:

1. EMLE: full EMLE embedding scheme, including models of molecular electronic density and polarization of the ML part
2. EMLE-NoPol (no polarization): EMLE scheme but without the polarization component
3. EMLE-Mech (mechanical): as “no polarization” but the atomic electronic densities are collapsed to the nucleus
4. MM: atomic partial charges taken from the MM force field and stay fixed during the simulation

The results for gas phase, water, and HSA protein simulations are shown in Table 1, where we report excitation energies and transition dipole moments computed for the L_a_ and L_b_ states of 3MI. In Figure 2, we then report the corresponding errors with respect to the benchmark data derived from ωB97X Born–Oppenheimer MD simulations. The agreement of transition energies and dipoles derived from ANI-2x ML-based simulations in the gas phase is striking, with errors <0.01 eV and <0.01 D for energies and dipoles, respectively. In comparison, the simulations based on the Amber ff14SB force field introduce significant absolute errors ∼0.06–0.14 eV and ∼0.15–0.22 D. The gas phase results clearly indicate that ANI-2x is superior to the employed MM force field and provides values close to the ones obtained at the reference DFT level of theory.

**Figure 2:**
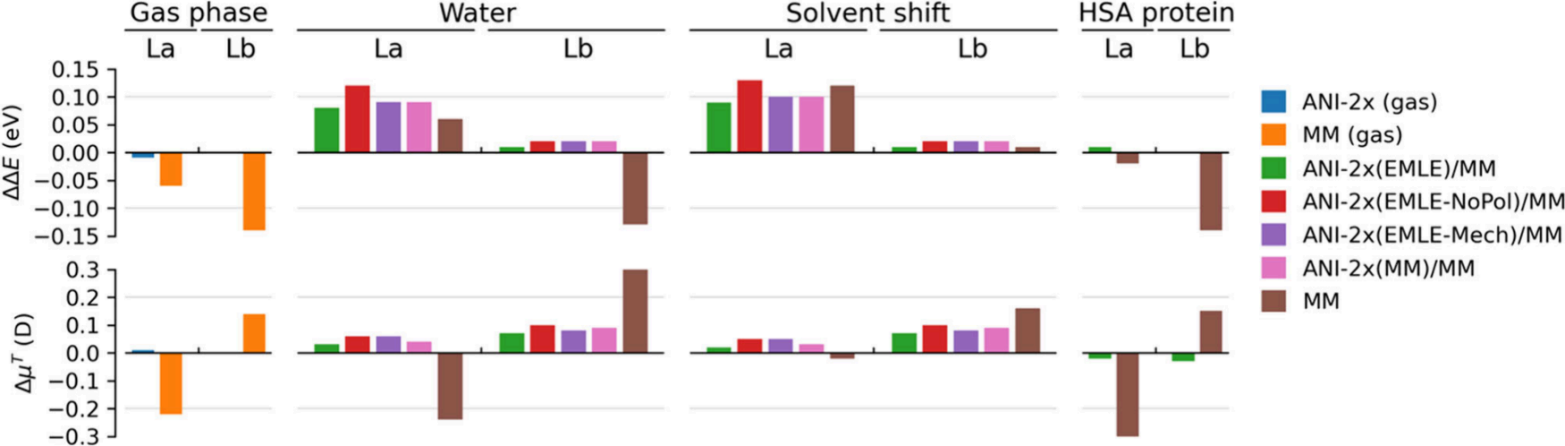
Deviation of mean QM/MMPol TD-ωB97X excitation energies and transition dipole moments of 3-methyl-indole derived from ML and MM-based MD trajectories with respect to benchmark ωB97X Born–Oppenheimer MD simulations. Top (left to right): excitation energies in the gas phase, excitation energies in aqueous solution, gas to water solvent shifts in excitation energies, and excitation energies in HSA. Bottom (left to right): transition dipoles in the gas phase, transition dipoles in aqueous solution, gas to water solvent shifts in transition dipoles, and transition dipoles in HSA.

**Table 1:** Mean Values of QM/MMPol TD-ωB97X Excitation Energies (eV) and Transition Dipole Moments (D) of 3-Methyl-Indole Derived from QM, MM and ML-Based MD Trajectories

	MD	Δ*E* L_a_	μ^T^ L_a_	Δ*E* L_b_	μ^T^ L_b_
gas phase	ωB97X	5.29	2.11	5.14	1.51
	MM	5.23	1.89	5.00	1.65
	ANI-2x	5.28	2.12	5.14	1.51
solvated	ωB97X/MM	5.10	2.20	5.13	1.54
	MM	5.16	1.96	5.00	1.84
	ANI-2x(EMLE)	5.18	2.23	5.14	1.61
	ANI-2x(EMLE-NoPol)	5.22	2.26	5.15	1.64
	ANI-2x(EMLE-Mech)	5.19	2.26	5.15	1.62
	ANI-2x(MM)	5.19	2.24	5.15	1.63
HSA protein	ωB97X/MM	5.17	2.30	5.12	1.76
	MM	5.15	1.95	4.98	1.91
	ANI-2x(EMLE)	5.18	2.28	5.12	1.73

The same trend is maintained in water and protein simulations—ANI-2x with EMLE embedding improves the prediction of both excitation energies and transition dipoles. The only exception is the excitation energy of the L_a_ state in water, where MM benefits from error cancelation and provides a value that is slightly closer to the DFT reference compared to EMLE­(ANI-2x). Indeed, this trend is not observed for the dipole, in which MM shows a considerable error of −0.24 eV for L_a_, and in the HSA protein environment both excitation energies and dipoles for the L_a_ state are nicely reproduced by ANI-2x­(EMLE). Overall, the ML/MM simulations show an excellent behavior. In all cases, they reproduce transition dipole moments with errors <0.1 D, compared to values ∼0.15–0.35 eV observed in MM data. In terms of excitation energies, in water, the energy of the L_b_ state is also nicely reproduced with an error ∼0.02 eV, whereas for L_a_ the errors are somewhat larger in the range 0.08–0.12 eV, as previously discussed. In the HSA protein, however, both L_a_ and L_b_ excitation energies are nicely reproduced with errors below 0.01 eV.

For all observables in water solution, inclusion of polarization in the full EMLE embedding scheme provides a minor, but non-negligible, improvement of the predictions. In water, however, polarization is not expected to have a significant impact on the generated geometries, but this could change in complex biological environments, such as in the HSA protein. Tryptophan indeed establishes frequent cation−π interactions with Lys or Arg residues, an interaction known to depend heavily on the polarization of the indole ring.^50–53^ Thus, inclusion of polarization could be important to describe the high sensitivity of Trp spectroscopic properties to the surrounding environment. Polarization is indeed important in a variety of key biological processes, and extensive efforts are devoted for example to the development of polarizable force fields in molecular simulations.^54^ In our case, we address the properties of Trp214 in HSA, which establishes a cation−π interaction with Lys199 and interacts with nearby Arg218, as shown in Figure 3. Our full EMLE embedding results in HSA lead to an excellent agreement with the data derived from benchmark QM/MM MD simulations, in terms of both excitation energies and transition dipole moments, suggesting EMLE can handle the polarization exerted by those cationic residues on Trp adequately.

**Figure 3:**
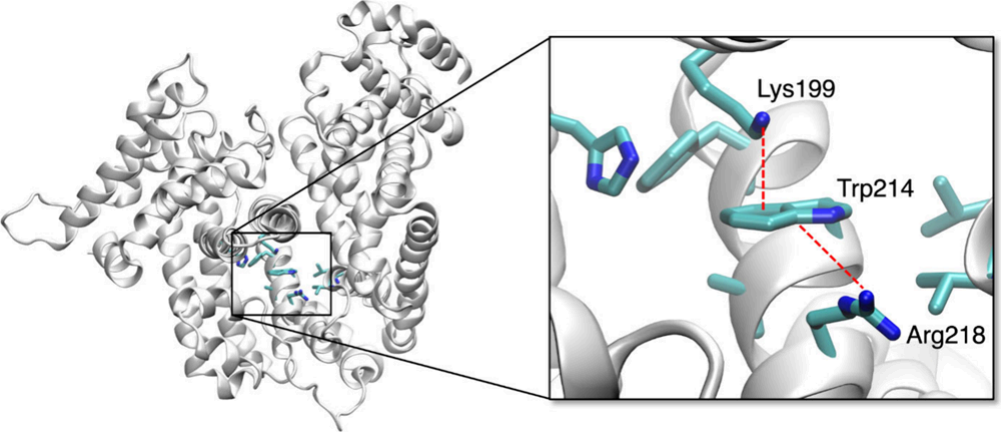
Arrangement of Trp214 in the HSA protein in the last frame of a QM/MM MD replica. Trp214 establishes a cation−π interaction with Lys199 and interacts with nearby Arg218.

Compared to experimental data, our QM/MMPol TD-ωB97X calculations correctly predict that L_b_ is the lowest excited state in the gas phase, although they lead to overestimated energies and an energy gap between L_a_ and L_b_ that is too small. This behavior for indole has been previously observed for long-range corrected functionals,^35,55^ with hybrid and meta-GGA functionals leading to an incorrect order of the states, this latter point also confirmed by our TD-B3LYP data (see Supporting Information). Beyond this, the TD-B3LYP results are qualitatively very similar to those based on TD-ωB97X, supporting the fact that ANI-2x with EMLE embedding dramatically improves the prediction of excitation energies and transition dipoles.

On the other hand, the computed solvatochromic shifts between gas phase and water solution indicate that L_a_ is strongly stabilized by the polar environment compared to L_b_, a well-known behavior leading to the level inversion mechanism often invoked to explain Trp properties in biosystems.^35^ In this case, solvent shifts for L_a_ obtained with ML/MM underestimate those based on QM/MM. However, in the HSA protein we observe a similar strong stabilization of the L_a_ state compared to L_b_, which is nicely reproduced by ML/MM results.

Excitation energies typically depend on the choice of the QM method, and *ad hoc* corrections can be applied to eventual systematic errors. Much more challenging is, however, to describe the coupling between excitations and vibrations that are encapsulated in the SDs. In this task, the improvement introduced by ML compared to MM is especially evident, in the gas phase, in water solution, and in HSA, as shown in Figure 4. In a vacuum, the SD of L_a_ is characterized by two large peaks at ∼1400 and ∼1700 cm^–1^, whereas for L_b_ the SDs still display the peak at ∼1400 cm^–1^ but lack the one at ∼1700 cm^–1^. These features are beautifully quantitatively reproduced for both states in all frequency ranges, with only the intensity of the peak at ∼1700 cm^–1^ in L_a_ being slightly underestimated. This result is in stark contrast with MM data, which miss the position of the main peaks and instead lead to high-frequency peaks at ∼1850 cm^–1^ and ∼1670 cm^–1^ in the L_a_ and L_b_ SDs, respectively.

**Figure 4:**
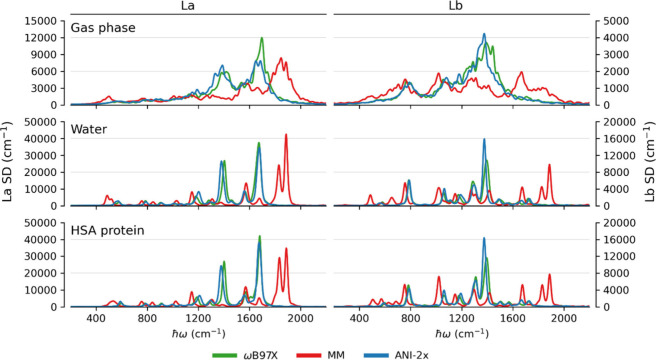
Comparison of spectral densities derived for the L_a_ and L_b_ states of 3-methylindole. ANI-2x stands for pure ANI-2x for the gas phase and ANI-2x­(EMLE) for the systems in water and in the HSA protein environment.

In a water solution or in the HSA environment, indole vibrations are modulated by the surrounding solvent and protein motions, an interplay that cannot be tackled by methods based on normal modes. For 3MI, this leads to some visible changes in the structure of the SD and sharper peaks compared to the gas phase, as shown in Figure 4. In this case, again, the performance of ML-based simulations based on ANI-2x with EMLE embedding is striking, reproducing almost quantitatively the SDs at all frequency ranges, whereas MM SDs again miss the main peaks and lead to overestimated high-frequency features. The inclusion of polarization in the full EMLE embedding scheme in this case provides minor variations in the intensity of the peaks (see Figure S2 in the Supporting Information).

To understand the basis behind the observed differences between ANI-2x­(EMLE) and MM predictions, we compared the geometries of 3MI sampled along MM and ANI-2x­(EMLE)/MM MD trajectories in water solution to the reference geometries from the ωB97X/MM MD. Figure 5 shows the absolute deviations of the mean values of all bonds, angles, and dihedrals present in the MM topology of 3MI. Clearly, ANI-2x­(EMLE)/MM results in a conformational ensemble much closer to QM/MM than the one provided by MM. Notably, the bonds and angles with the largest deviations at the MM level play an important role in the vibrational normal mode at ℏω ≈ 1700 cm^–1^, characterized by in-plane bendings and C–C and C–N stretchings that lead to ring breathing. This explains, thus, why this vibration, which displays the most intense peak in the observed ωB97X and ANI-2x SDs (Figure 4), is missed by MM.

**Figure 5:**
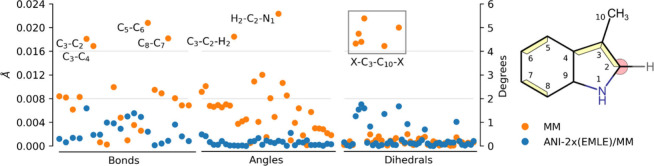
Absolute deviations of average bond, angle, and dihedral angle values obtained from ANI-2X­(EMLE)/MM simulations of 3MI in water compared to reference ωB97X/MM values. Bonds and angles with the largest deviations at the MM level are highlighted on the 3MI structure on the right: yellow for the bonds and pink for the angles. All of the dihedrals with largest deviations correspond to the rotation of the methyl group (C_3_–C_10_ bond).

On the other hand, the six dihedral angles with largest errors all correspond to the rotation of the methyl group. As shown on Figure S11, MM fails to describe the rotational barrier present at the ωB97X level and results in an almost flat distribution of the dihedral angles, whereas ANI-2x perfectly reproduces the reference distribution.

For completeness, we have also checked the impact of the potential on the solvent structure around 3MI by comparing radial distribution functions of the water oxygens around selected atoms of 3MI. We have not observed any notable differences (Figure S12). Therefore, we conclude that the improvement provided by EMLE compared to MM is fully explained by the impact on the conformational ensemble of 3MI, not the surrounding solvent.

To shed light on the sharpening of the SD features in the condensed phase, in Figures S9 and S10 we compare the SDs derived from MD simulations in the gas phase and in water to those computed from normal modes using the VG method (in vacuum). The SDs obtained using both approaches in the gas phase are in good agreement, once a broadening with a HWHM of 35 cm^–1^ is applied to the Lorentzian line shape used to build the VG SD. In contrast, the water SDs derived from MD trajectories are in better agreement with the VG SDs derived adopting a HWHM of 15 cm^–1^. The broader features in the vacuum SDs then explain why the SDs are less smooth than those obtained in solution, as they probably require larger samplings to be well converged.

Overall, our results show a dramatic improvement of ML/MM over pure MM simulations. Indeed, important discrepancies between MM and QM-derived SDs have already been observed in systems like pigment–protein complexes.^22^ For example, a shifted equilibrium position of the PES with respect to the QM one can lead to exaggerated fluctuations and overestimated peaks,^20^ and an SD derived from classical MD will feature peaks at the MM frequencies rather than at frequencies corresponding to the QM method used for excited states, leading to errors in the position of the SD peaks and a redistribution of their intensities.^2^ This is reflected in the fact that SDs based on MM are typically more sensitive to the choice of force field than to the QM method used for excited states.^56^ Overall, our results indicate that ML/MM simulations explore a PES that is highly consistent with the QM/MM one. Of course, this agreement is helped by the fact that we run dynamics with ωB97X/6-31G­(d), the same level of theory used to train ANI-2x on energies and forces of small molecules, and that 3MI is a relatively rigid system, facilitating the training of an MLP. Nevertheless, our results here show that ANI-2x combined with EMLE electrostatic embedding is also able to reproduce with high fidelity the PES of a small molecule explored in aqueous solution and in a protein environment.

Performance-wise, ML potentials provide significant reductions in computational cost, even compared to the most efficient QM codes. For consistency with the reference QM/MM calculations, the ML/MM MD simulations presented in this work were performed with the sander interface of the emle-engine, for which the computational bottleneck is the socket-based communication between the sander and the emle-server code. Nevertheless, even in this case, the performance of ML/MM MD is ∼200 ps/day, 300-fold higher than that of sander+ORCA running on a single core (∼ 0.5 ps/day). However, such a comparison is not fair, since the emle engine employs a graphical processing unit (GPU) that has much more computing power. For a fair comparison and to get an estimate of “best-case scenario” for both QM/MM and ML/MM, we have performed additional QM/MM calculations using QUICK, a GPU-accelerated QM package, and an ML/MM calculations using the OpenMM interface with emle-engine available in the Sire framework.^57^ Again, resulting ML/MM simulations are ∼300-fold faster (6 ns/day) than their QM/MM counterpart (19 ps/day). A summary of the performance obtained with different codes is provided in Table S2.

Overall, we conclude that the combination of ML potentials with the EMLE electrostatic embedding scheme is optimally suited for studying the interrelation between the photophysics of biological chromophores and the multiple time scales of biomolecular motion. We envision relevant future applications in photobiology, for example, the investigation of the role of vibronic coupling in coherent energy transfer or the role of the protein in tuning pigment energies in photosynthetic light harvesting complexes. Alternatively, in cases where long time scales need to be sampled, it will be possible to use accurate simulations based on ML/MM to train ML models directly designed to derive properties from structure, for example the recent method proposed by Cignoni and co-workers to predict excitonic Hamiltonians of light harvesting complexes.^58^

## Supplementary Material





## Data Availability

The data that support the findings of this work are publicly available at https://github.com/kzinovjev/emle-qmmmpol

## Notes

Figures of autocorrelation functions; spectral densities and excitation energy trajectories from TD-ωB97X and TD-B3LYP calculations; figure and table with TD-B3LYP mean excitation energies, transition dipoles, and corresponding errors; figures of dihedral angles and radial distribution functions; and table with performances of QM/MM and ML/MM with different implementations (PDF)
Transparent Peer Review report available (PDF)
