# Supplementary material for: Improved Description of Environment and Vibronic Effects with Electrostatically Embedded ML Potentials
Source: J Phys Chem Lett. 2025 Jan 13;16(3):774–81. doi: 10.1021/acs.jpclett.4c02949 (PMC12216233; doi:10.1021/acs.jpclett.4c02949)
Supplement: Supplementary file 1 [file jz4c02949_si_001.pdf]

Improved description of environment and vibronic effects with electrostatically embedded ML potentials

Supporting Information

Kirill Zinovjev[1], Carles Curutchet[2,3**]*

[1]Departamento de Química Física, Universidad de Valencia, 46100, Burjassot (Spain)

[2]Departament de Farmàcia i Tecnologia Farmacèutica, i Fisicoquímica, Facultat de Farmàcia i Ciències de l'Alimentació, Universitat de Barcelona (UB), 08014, Barcelona, Spain

[3]Institut de Química Teòrica i Computacional (IQTCUB), Universitat de Barcelona (UB), 08028, Barcelona, Spain

Corresponding Authors

*kirill.zinovjev@uv.es, **carles.curutchet@ub.edu

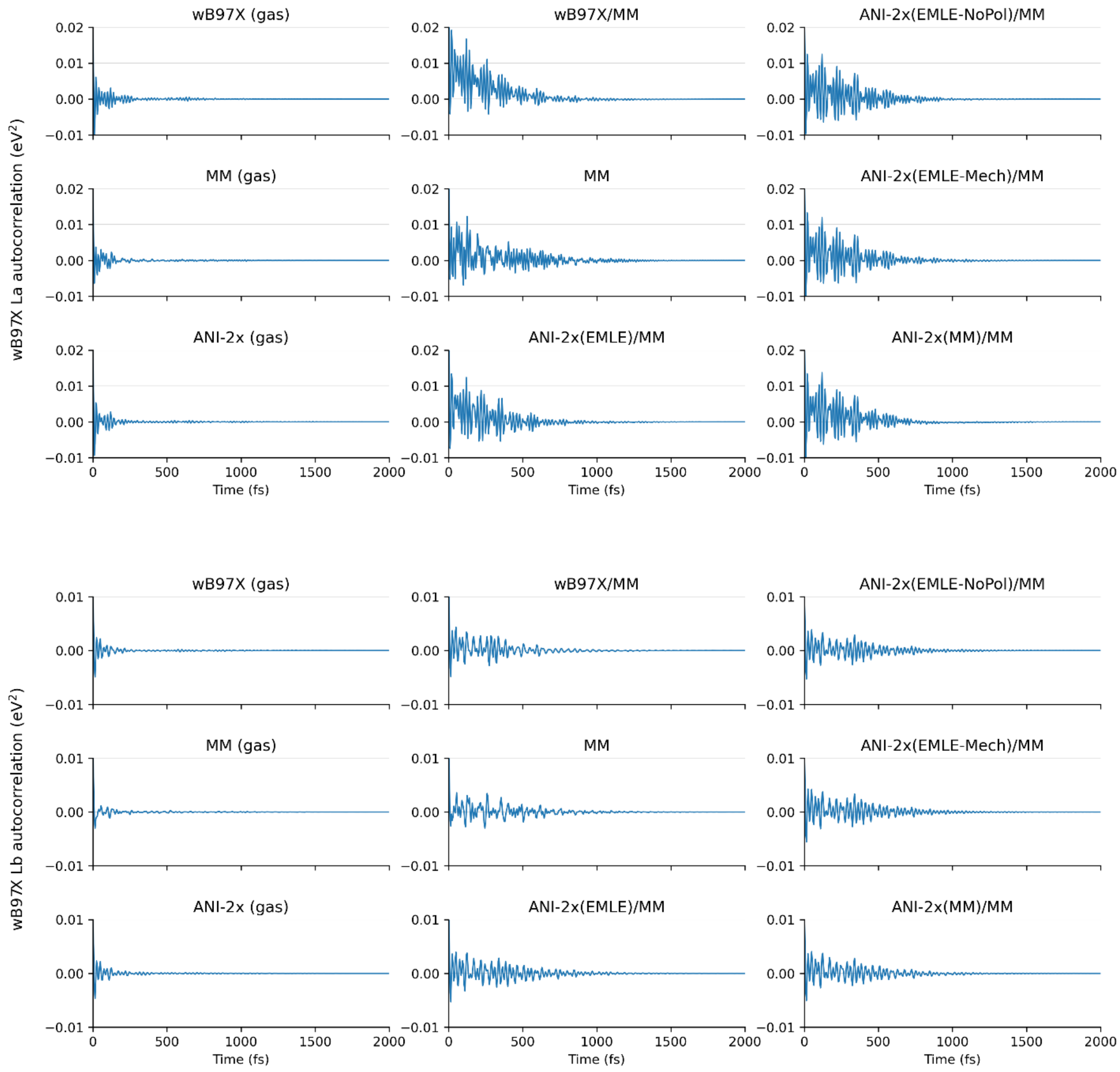

Figure S1. Autocorrelation functions of QM/MMPol TD-ωB97X excitation energies of the L_a (top) and L_b (bottom) states of 3-methyl-indole derived from QM, ML and MM-based MD trajectories.

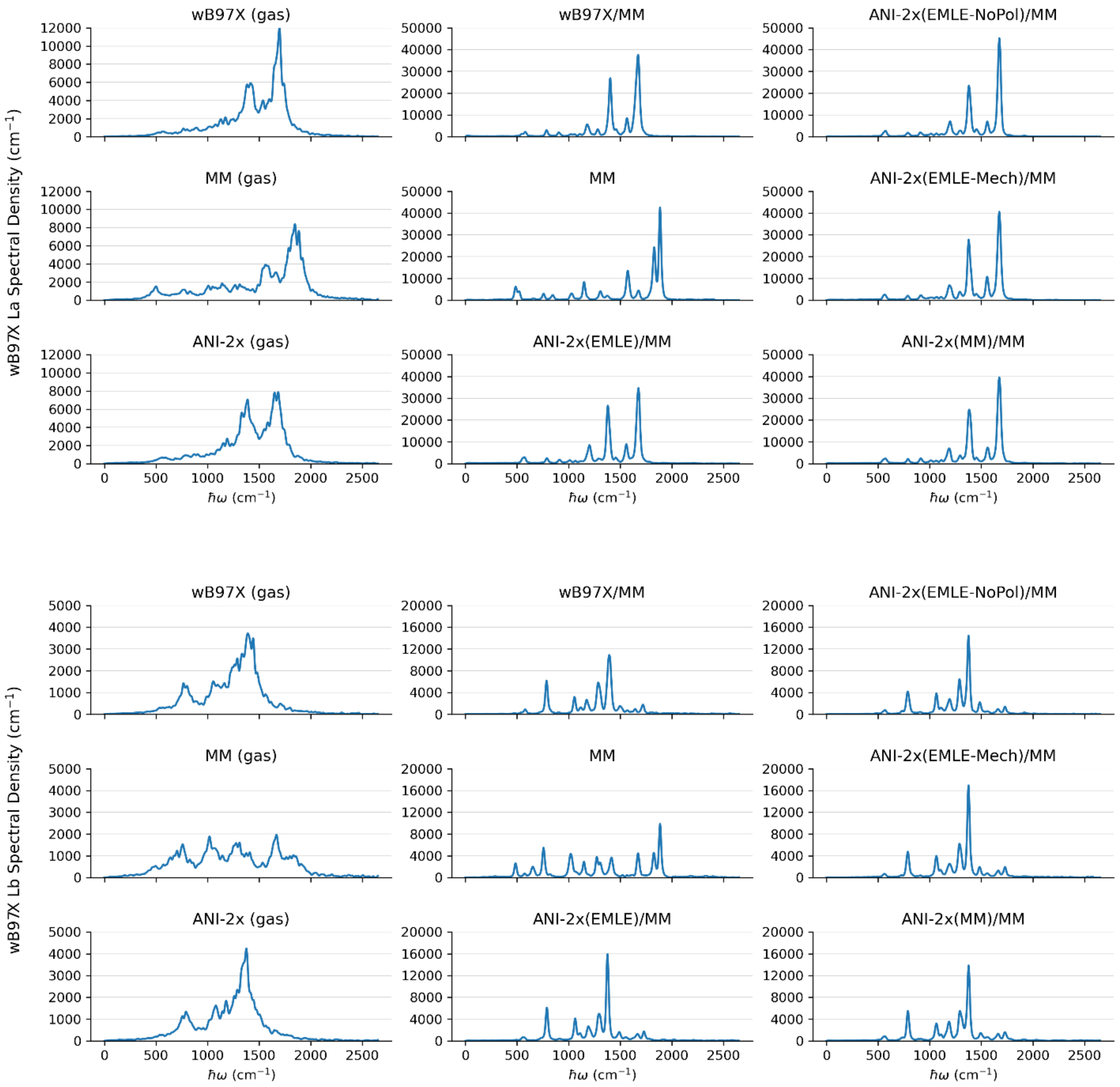

Figure S2. Spectral densities computed from QM/MMPol TD-ωB97X excitation energies of the L_a (top) and L_b (bottom) states of 3-methyl-indole derived from QM, ML and MM-based MD trajectories.

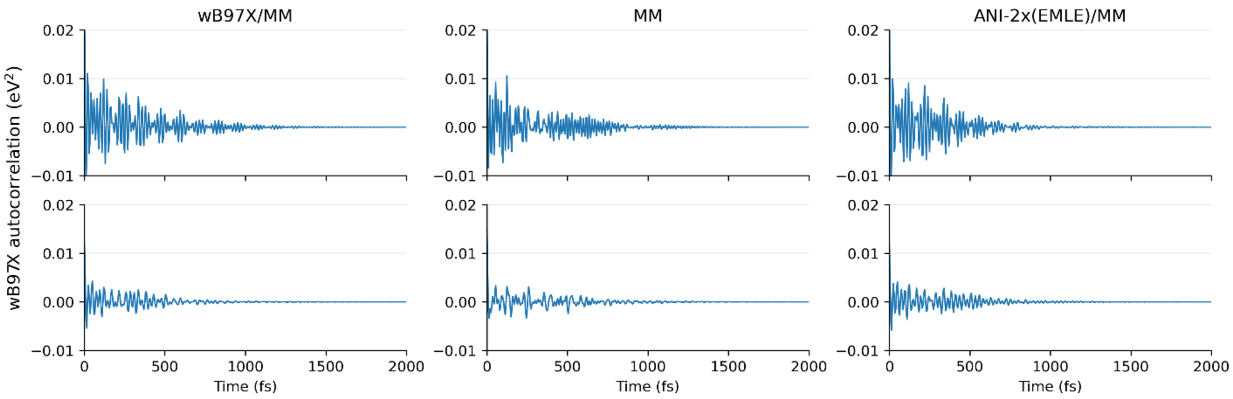

Figure S3. Autocorrelation functions of QM/MMPol TD-ωB97X excitation energies of the L_a (top) and L_b (bottom) states of 3-methyl-indole in HSA derived from QM, MM and ML-based MD trajectories.

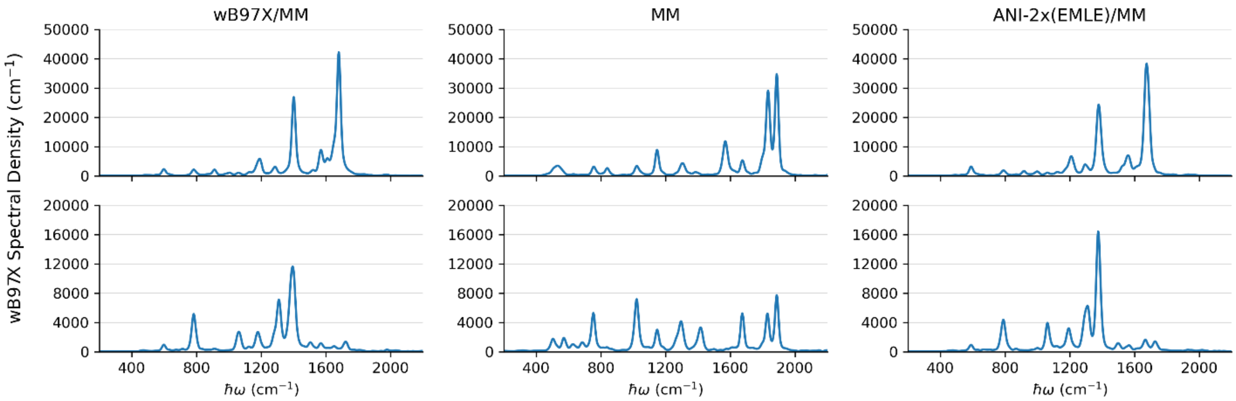

Figure S4. Spectral densities computed from QM/MMPol TD-ωB97X excitation energies of the L_a (top) and L_b (bottom) states of 3-methyl-indole in HSA derived from QM, MM and ML-based MD trajectories.

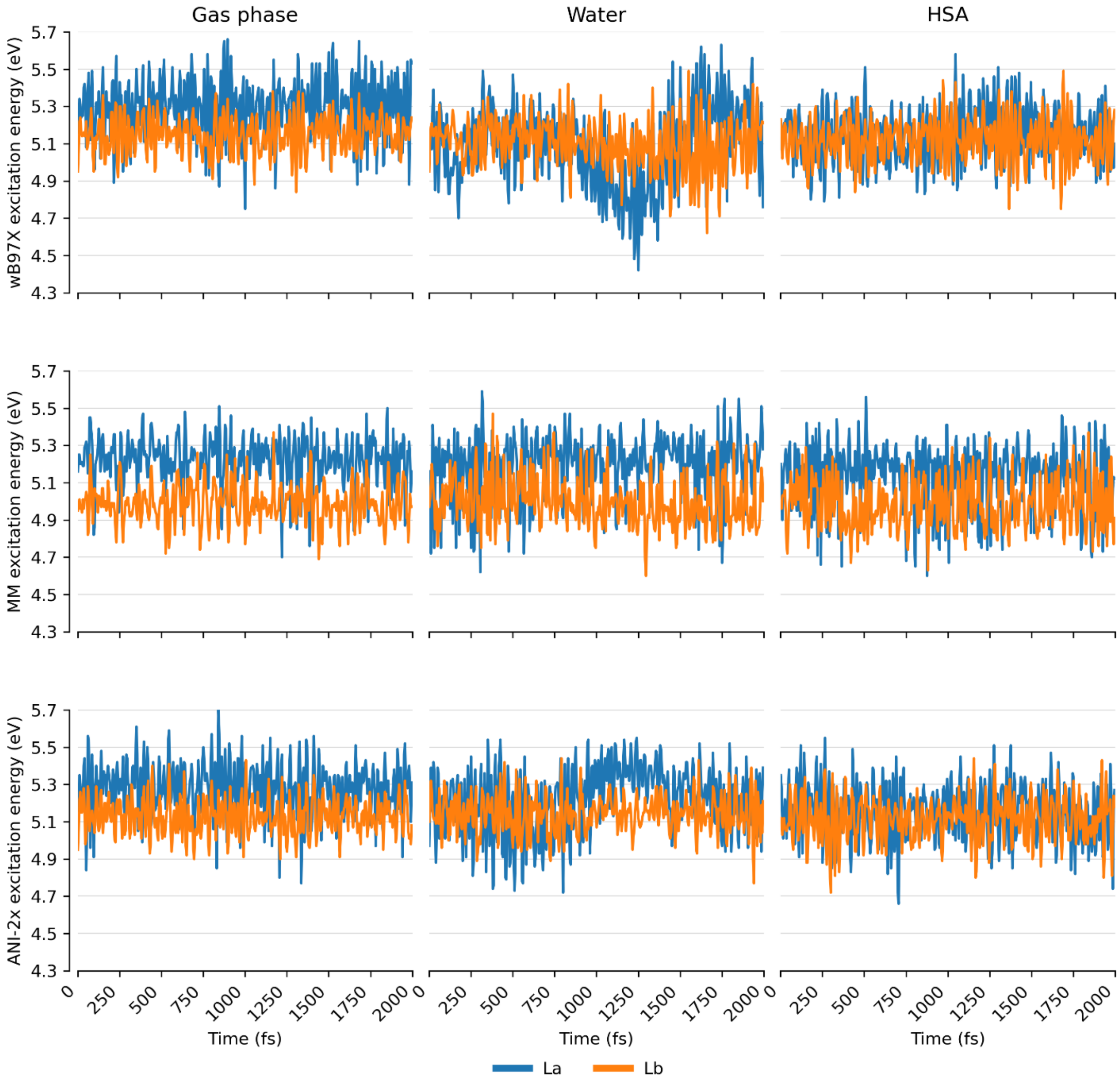

Figure S5. Examples of trajectories of QM/MMPol TD-ωB97X excitation energies of the L_a and L_b states of 3-methyl-indole in gas phase, water and HSA derived from QM, MM and ML-based MD simulations.

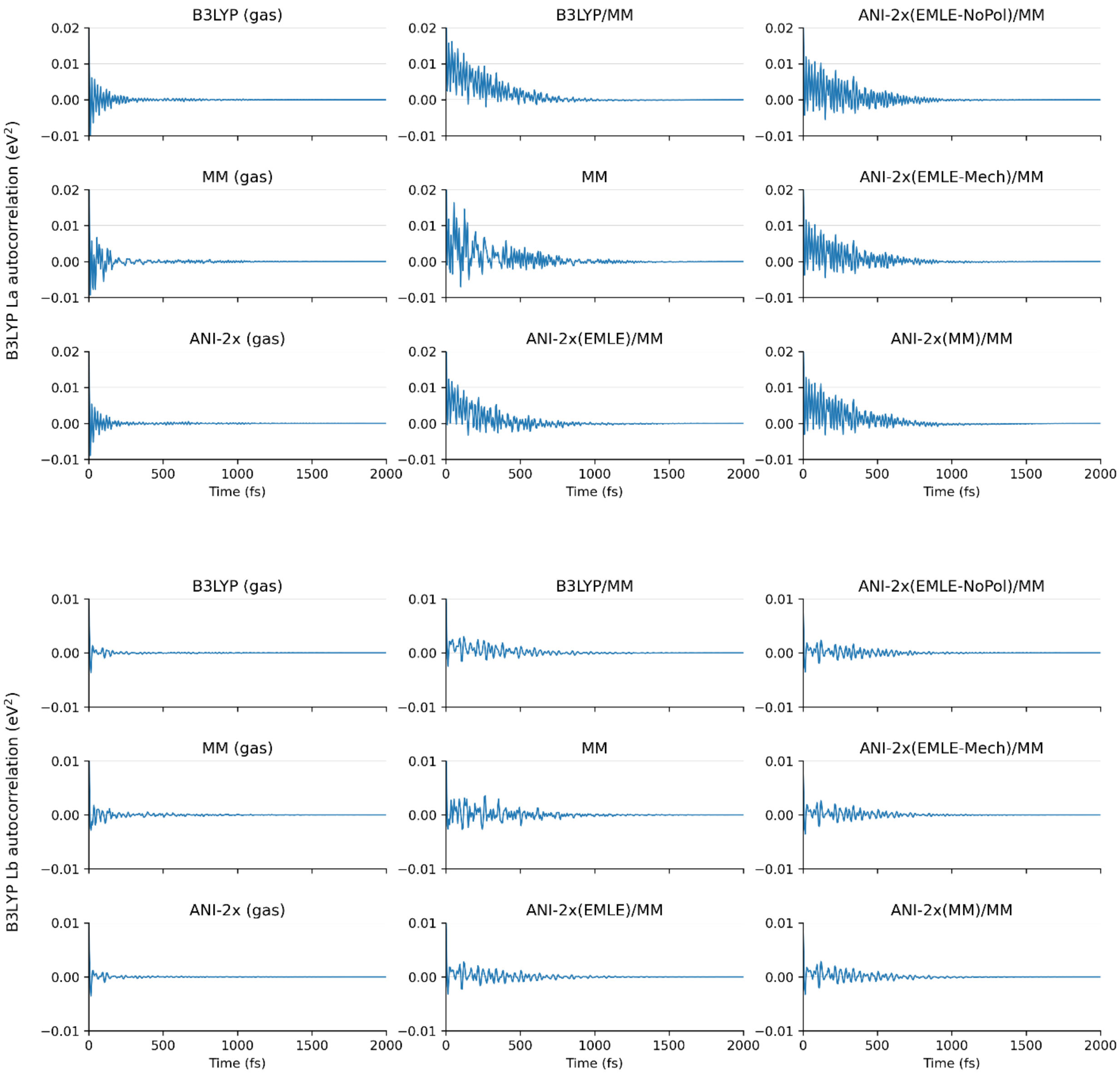

Figure S6. Autocorrelation functions of QM/MMPol TD-B3LYP excitation energies of the L_a (top) and L_b (bottom) states of 3-methyl-indole derived from QM, ML and MM-based MD trajectories.

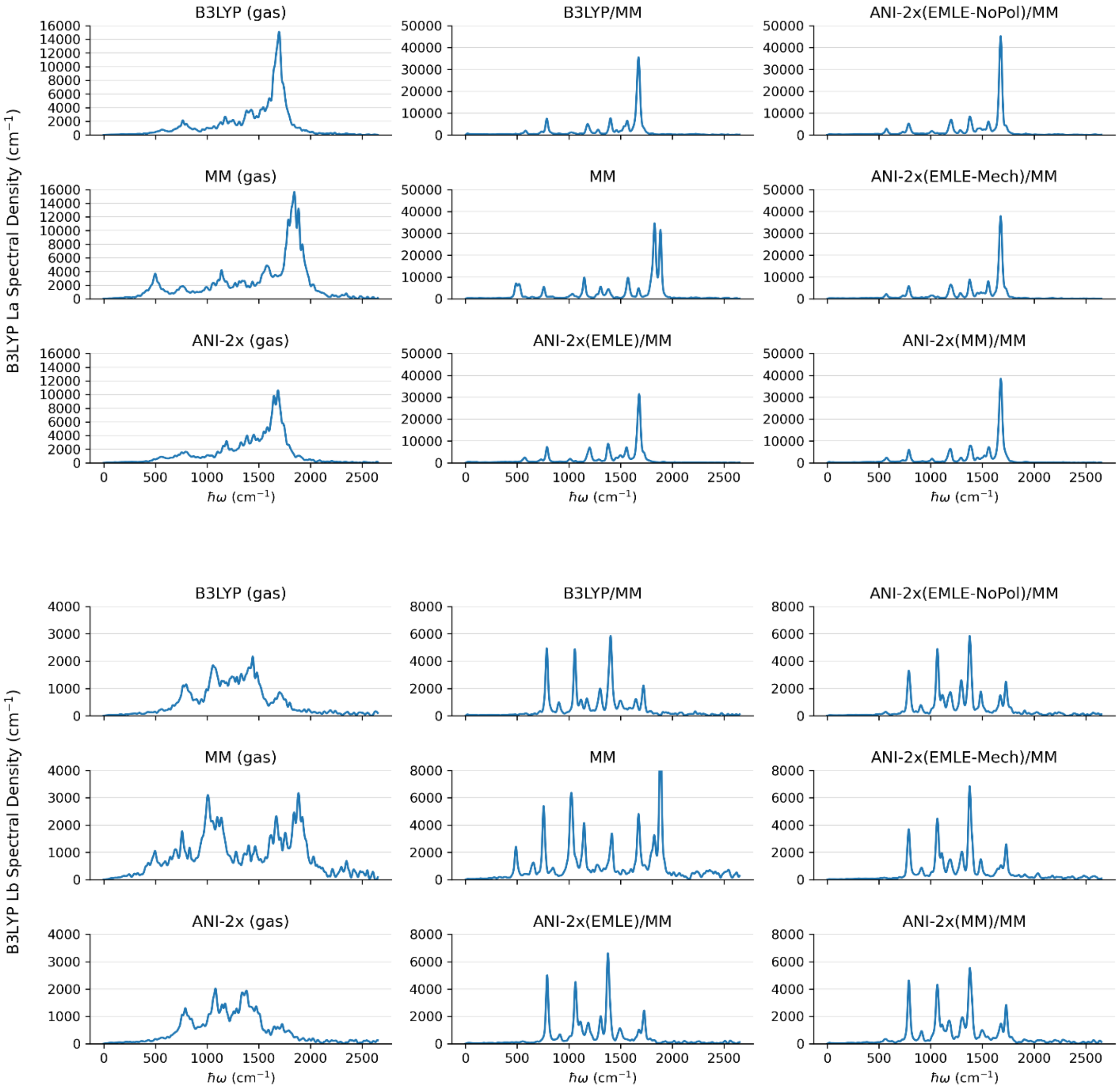

Figure S7. Spectral densities computed from QM/MMPol TD-B3LYP excitation energies of the L_a (top) and L_b (bottom) states of 3-methyl-indole derived from QM, ML and MM-based MD trajectories.

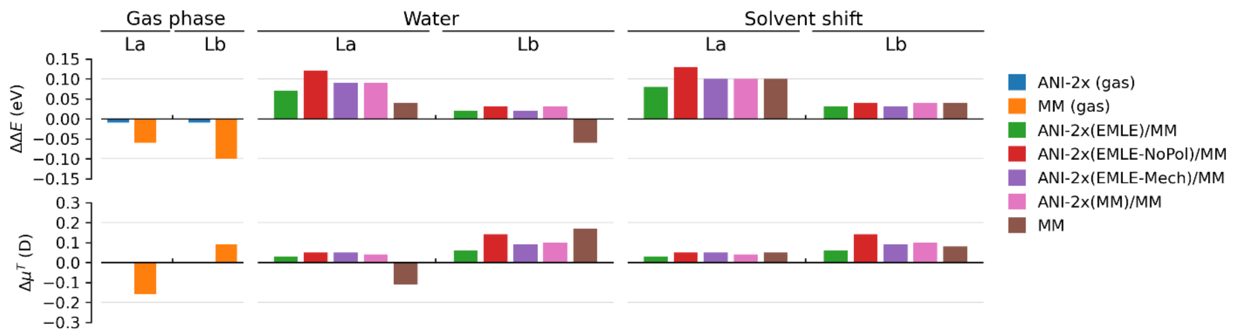

Figure S8. Errors in QM/MMPol TD-B3LYP excitation energies and transition dipole moments of 3-methyl-indole derived from ML and MM-based MD trajectories with respect to benchmark ωB97X/MM Born-Oppenheimer MD simulations.

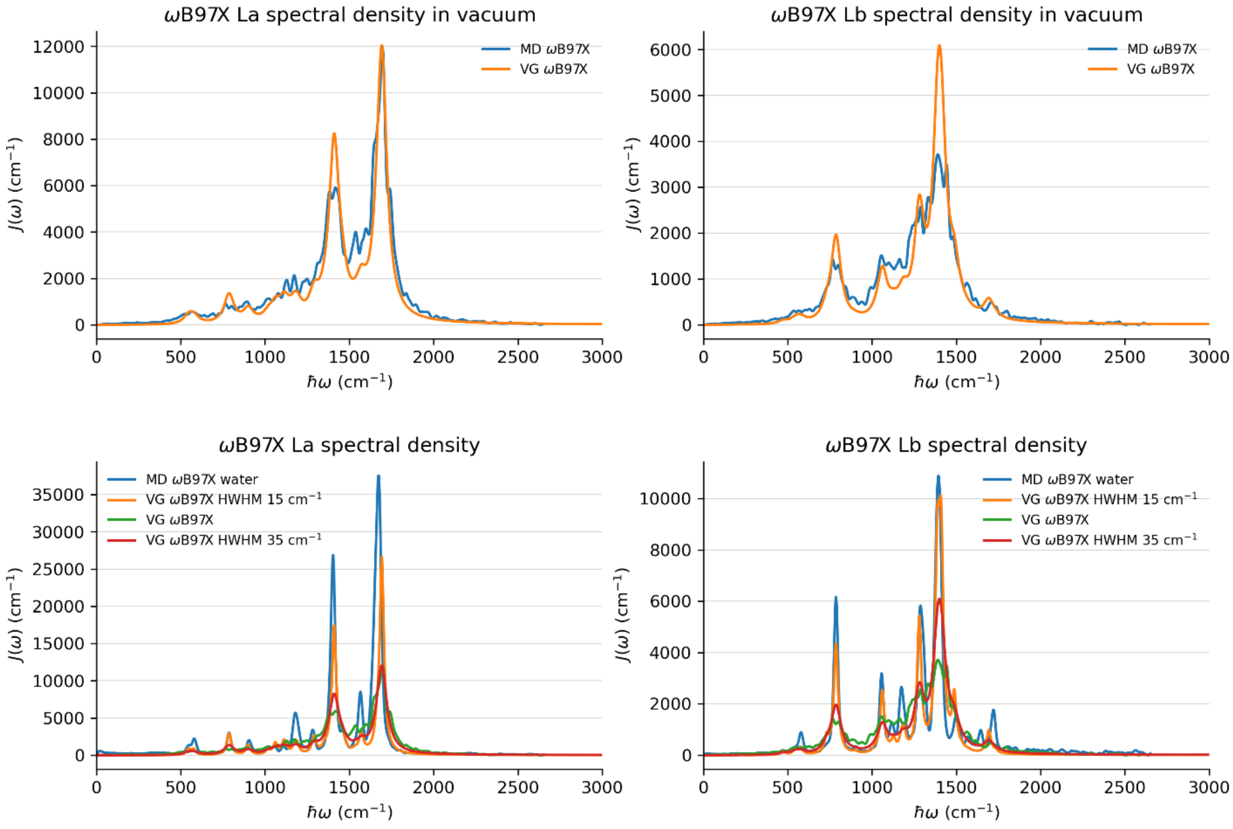

Figure S9. Spectral densities computed from TD-ωB97X excitation energies of the L_a and L_b states of 3-methyl-indole in gas phase derived from QM calculations using the vertical gradient method (broadening with a HWHM of 35 cm^{-1}) or the dynamic approach based on QM MD trajectories. In the lower panel, we compare the spectral densities obtained using the dynamic approach based on MD trajectories in water and in gas phase with VG spectral densities obtained using different values for the broadening parameter HWHM used in the Lorentzian lineshape.

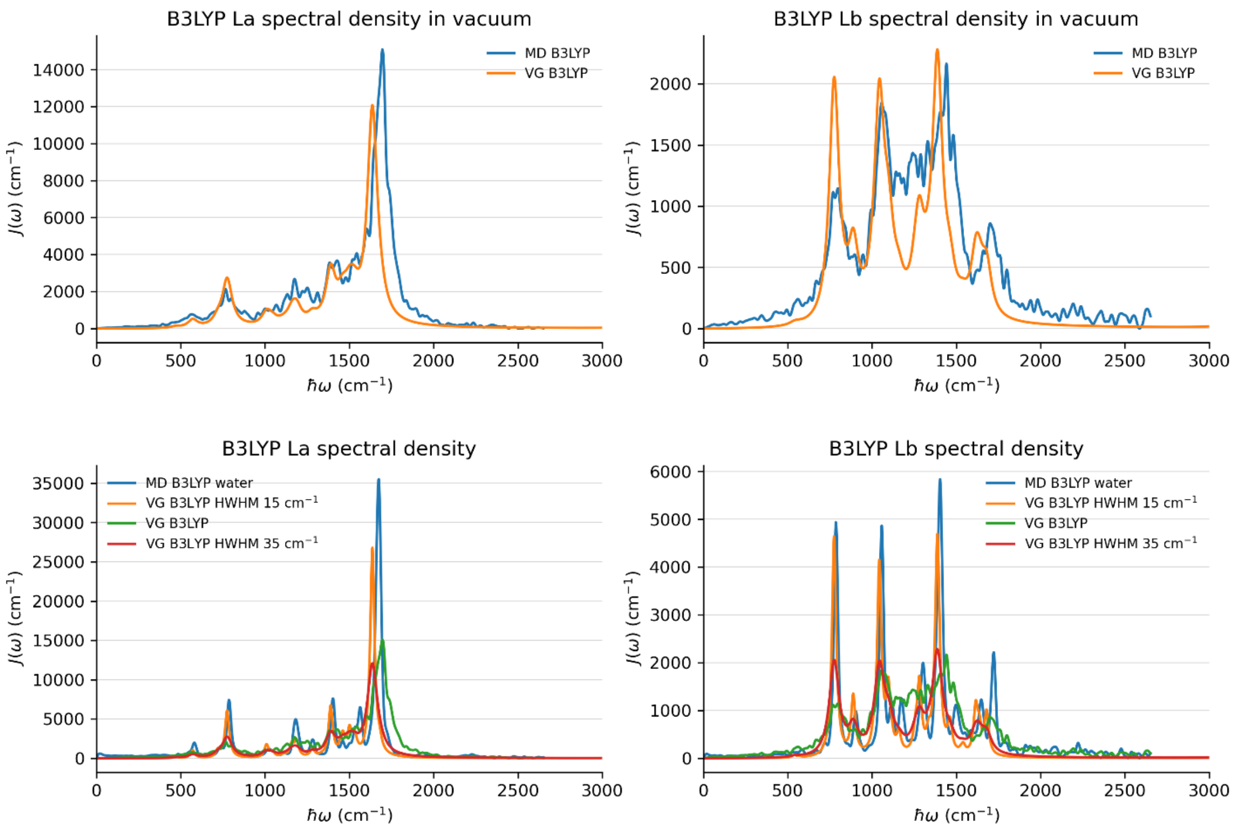

Figure S10. Spectral densities computed from TD-B3LYP excitation energies of the L_a and L_b states of 3-methyl-indole in gas phase derived from QM calculations using the vertical gradient method (broadening with a HWHM of 35 cm^{-1}) or the dynamic approach based on QM MD trajectories. In the lower panel, we compare the spectral densities obtained using the dynamic approach based on MD trajectories in water and in gas phase with VG spectral densities obtained using different values for the broadening parameter HWHM used in the Lorentzian lineshape.

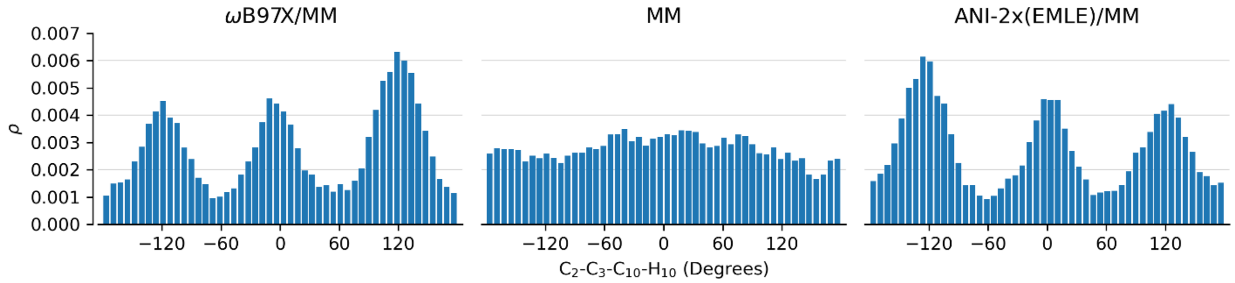

Figure S11. Distribution of one of the dihedral angles corresponding to the rotation of the methyl group in 3-methyl-indole derived from QM, MM and ML-based MD trajectories in water solution.

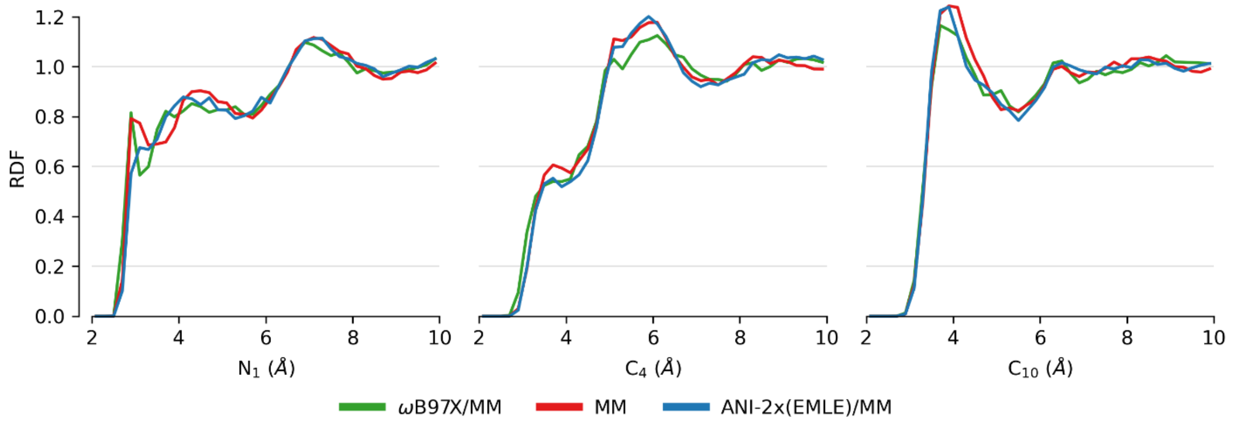

Figure S12. Radial distribution functions of water oxygen atoms around atoms N_1, C_4 and C_{10} of 3-methyl-indole derived from QM, MM and ML-based MD trajectories in water solution.

Table S1. QM/MMPol TD-B3LYP excitation energies (eV) and transition dipole moments (D) of 3-methyl-indole derived from QM, ML and MM-based MD trajectories.

	MD	$\Delta E\ L_a$	$\mu^T\ L_a$	$\Delta E\ L_b$	$\mu^T\ L_b$
gas phase	ωB97X	4.74	1.78	4.92	1.08
	MM	4.68	1.62	4.82	1.17
	ANI-2x	4.73	1.78	4.91	1.08
solvated	ωB97X/MM	4.55	1.91	4.89	0.95
	MM	4.59	1.80	4.83	1.12
	ANI-2x(EMLE)	4.62	1.94	4.91	1.01
	ANI-2x (EMLE-NoPol)	4.67	1.96	4.92	1.09
	ANI-2x (EMLE-Mech)	4.64	1.96	4.91	1.04
	ANI-2x(MM)	4.64	1.95	4.92	1.05

Table S2. Performance of QM/MM and ML/MM with different implementations.

Implementation	Performance (ps/day)
QM/MM: sander+ORCA (Intel Xeon Platinum 8480+, 1 core)	0.56
QM/MM: sander+ORCA (Intel Xeon Platinum 8480+, 8 cores)	2.8
QM/MM: sander+QUICK (NVIDIA H100)	18.9
ML/MM: sander+emle-engine (NVIDIA H100)	206
ML/MM: OpenMM+sire+emle-engine (NVIDIA H100)	6000
