# Supplementary material for: Improved Description of Environment and Vibronic Effects with Electrostatically Embedded ML Potentials
Source: J Phys Chem Lett. 2025 Jan 13;16(3):774–81. doi: 10.1021/acs.jpclett.4c02949 (PMC12216233; doi:10.1021/acs.jpclett.4c02949)
Supplement: Supplementary file 2 [file jz4c02949_si_002.pdf]

jz-2024-02949z.R1

Name: Peer Review Information for "Improved description of environment and vibronic effects with electrostatically embedded ML potentials"

First Round of Reviewer Comments

Reviewer: 1

Comments to the Author

Manuscript ID: jz-2024-02949z

Title: "Improved description of environment and vibronic effects with electrostatically embedded ML potentials" General Assessment:

The manuscript presents a novel approach, combining ML/MM and QM/MM simulations to compute spectral densities and excited-state energies efficiently. The authors employ the EMLE engine with the ANI-2x potential to sample the ground-state PES, followed by QM/MMPol single-point calculations. This protocol speeds up the process while maintaining accuracy, and the results demonstrate that ML/MM predictions closely match QM/MM results, outperforming MM-based simulations. The paper is well-written, the topic is relevant and I consider it suitable for publication in The Journal of Physical Chemistry Letters, after addressing several key issues.

Major comments:

1) Extension to a More Complex System

While the chosen system (3-methyl-indole in aqueous solution) is appropriate for demonstrating the proof of concept, the complexity of biological environments -particularly in protein systems- should not be overlooked. The study would significantly benefit from testing the ML/MM implementation on a more complex system, such as an indole-containing residue (e.g., tryptophan) in a protein environment. Since the EMLE engine is integrated with Sander, it should be feasible.This could provide insights into how the method handles critical non-covalent interactions, such as π-π interactions, which are known to play a vital role in chromophore behavior within proteins. See doi.org/10.1021/jp3097378 for an example, and as the authors mention (page 11), polarization could be crucial in this context.

2) Structural Explanation for Ensemble Differences

The study demonstrates differences between the ML/MM, QM/MM, and MM ensembles, reflected in the calculated spectra. However, there is limited discussion on the molecular basis behind these differences. An analysis of geometrical parameters (i.e. radial distribution functions or key distances) could help clarify how ML/MM captures differences in the PES that MM cannot.

3) Computational Efficiency and Comparison with Other Methods

The manuscript mentions that ML/MM offers computational savings compared to QM/MM but lacks quantitative comparisons. There are GPU-accelerated QM/MM implementations that have demonstrated efficiency gains for sampling the ground-state PES, or even calculating spectra for small molecules. For instance, QUICK is available in AmberTools (doi.org/10.1021/acs.jcim.1c00169), and TD-DFT QM/MM simulations of indole in water have been successfully performed using another GPU-accelerated software (doi.org/10.1021/acs.jpca.0c06631). A comparison of the computational cost between the ML/MM method and other modern approaches, or at least an estimate of the time savings between ML/MM and QM/MM when sampling the ground-state PES, would offer readers a clearer understanding of the method's practical advantages and potential limitations.

Minor Comments:

- **Figure 2**: The resolution is too low.
- **Page 4, Line 3**: NMA is defined but never used again.
- **Page 4, Line 6**: The sentence “A more attractive...” is redundant with “A remedy for this problem...” on Page 3, Line 41.
- **Page 4, Lines 26, 50, 55**: QM, and MM, have not been defined previously. ● **Page 4, Line 55**: ML has not been defined previously (and maybe it should say MLP) ● **Page 5, Line 17**: *In vacuo* should be italicized.
- **Page 7, Line 14**: The basis set used to train ANI-2x is missing.
- **Page 7, Line 17**: *In vacuo* should be italicized.
- **Page 7, Line 40**: "The" Torchani package?
- **Page 8, Line 3**: Define La and Lb states for non-specialist readers; cutoff values for QM/MM and ML/MM are missing.
- **Page 8, Line 31**: Should it be “The solvated system”?
- **Page 12, Line 41**: There is a “.” before “whereas”; consider revising to “display” and “but lack the one at...”.
- **Page 13, Line 22**: PES has not been defined previously.
- **Page 13, Line 34**: Mention that 3-methylindole is a relatively rigid system when discussing PES consistency between ML/MM and QM/MM.

Reviewer: 2

Comments to the Author

In this work by Zinovjev and Curutchet, the authors present a comparison of excited state properties and spectral densities obtained from sampling configurations from either an MM force field trajectory or from a machine learned potential using the AN1-2x ML potential combined with an embedding scheme to account for solvent environment. The results show that energies and spectral densities agree well with those computed from a QM/MM trajectory, where the QM DFT method is the same wB97X functional used to train the AN1-2x ML potential. Overall, this seems like a very promising approach, and being able to reproduce the spectral density definitely showcases the abilities of the method. However, it is difficult to assess the comparison of the approach with the MM sampling, as there are no details given about the MM forcefield. Was it parametrized to use the same wB97X charges? Was it parametrized with other ab initio data (e.g. the Hessian)? Beyond this lapse in information, there are additional ways that the authors can improve the manuscript.

1) Figure 1 would benefit from a more detailed caption, with a) b) c) labels explaining each component.

2) In the paragraph beginning, "Here, we combine EMLE simulations...", I suggest modifying the sentence "We thus generated structures of 3MI..." to "We thus generated structures of 3MI solvated in water..."

3) It is unclear to me if there is any mixing of the La and Lb states during the trajectories. Can the authors add a plot to the SI with the La and Lb state energies as a function of time? Also, it would be good to give the specific details of how they identified each state, e.g. from alignment of the transition dipole with the ground state dipole?

4) Is the y-axis in Figure 2 a mean signed error? Similarly, is Table 1 showing average values?

5) In the spectral density figure, it seems that the gas phase spectral densities are much lower resolution. Were these from a long enough trajectory? I don't understand why they are much lower quality than the solvated results.

Author's Response to Peer Review Comments:

Dear Editor,

In the revised manuscript, we have made significant improvements based on the concerns raised by the reviewers. This includes additional calculations performed on an enzymatic system, as requested by Reviewer 1. We hope you will find the revised version suitable for publication in J. Phys. Chem. Lett.

Attached is a point-by-point response to both reviewers. For convenience, we also attach a PDF file of the manuscript highlighting all the introduced changes.

Reviewer 1

General Assessment:
The manuscript presents a novel approach, combining ML/MM and QM/MM simulations to compute spectral densities and excited-state energies efficiently. The authors employ the EMLE engine with the ANI-2x potential to sample the ground-state PES, followed by QM/MMPol single-point calculations. This protocol speeds up the process while maintaining accuracy, and the results demonstrate that ML/MM predictions closely match QM/MM results, outperforming MM-based simulations. The paper is well-written, the topic is relevant and I consider it suitable for publication in The Journal of Physical Chemistry Letters, after addressing several key issues.

We kindly thank the reviewer for the positive evaluation of our work. The answers to their comments are provided below.

Major comments:
1) Extension to a More Complex System
While the chosen system (3-methyl-indole in aqueous solution) is appropriate for demonstrating the proof of concept, the complexity of biological environments -particularly in protein systems- should not be overlooked. The study would significantly benefit from testing the ML/MM implementation on a more complex system, such as an indole-containing residue (e.g., tryptophan) in a protein environment. Since the EMLE engine is integrated with Sander, it should be feasible.This could provide insights into how the method handles critical non-covalent interactions, such as π-π interactions, which are known to play a vital role in chromophore behavior within proteins. See doi.org/10.1021/jp3097378 for an example, and as the authors mention (page 11), polarization could be crucial in this context.

As requested, in the revised manuscript we have performed additional simulations for a tryptophan residue in the human serum albumin (HSA) protein. This protein has a single tryptophan (Trp214), which establishes a cation-pi interaction with Lys199. In addition, it also interacts with another nearby cationic residue, Arg218, as shown in the new Figure 3. We found that in this case ANI-2x with EMLE embedding provides results even closer to the reference QM/MM calculations than in the case of water solution, whereas MM still leads to important errors. Thus, these results support the robustness of the method in this more challenging biological environment, indicating that polarization exerted by those cationic residues on Trp is well captured by EMLE. A discussion of these findings is now provided in the article.

2) Structural Explanation for Ensemble Differences
The study demonstrates differences between the ML/MM, QM/MM, and MM ensembles, reflected in the calculated spectra. However, there is limited discussion on the molecular basis behind these differences. An analysis of geometrical parameters (i.e. radial distribution functions or key distances) could help clarify how ML/MM captures differences in the PES that MM cannot.

In the revised manuscript we have systematically analyzed the differences between the conformational ensembles obtained at MM, ML/MM and reference QM/MM levels of theory in terms of average bonds, angles and torsions, as well as radial distribution functions. The results indeed provide an explanation for the superior performance of the ML/MM compared to MM. For example, the vibration near 1700 cm^{-1}, which is strongly coupled to the Trp La excited state as reflected by the peak in spectral densities, is characterized by in-plane bendings and C–C and C–N stretchings that lead to ring breathing, and those angles and bonds correspond to structural parameters which are particularly badly described by MM compared to ML.

3) Computational Efficiency and Comparison with Other Methods
The manuscript mentions that ML/MM offers computational savings compared to QM/MM but lacks quantitative comparisons. There are GPU-accelerated QM/MM implementations that have demonstrated efficiency gains for sampling the ground-state PES, or even calculating spectra for small molecules. For instance, QUICK is available in AmberTools (doi.org/10.1021/acs.jcim.1c00169), and TD-DFT QM/MM simulations of indole in water have been successfully performed using another GPU-accelerated software (doi.org/10.1021/acs.jpca.0c06631). A comparison of the computational cost between the ML/MM method and other modern approaches, or at least an estimate of the time savings between ML/MM and QM/MM when sampling the ground-state PES, would offer readers a clearer understanding of the method's practical advantages and potential limitations.

We thank the reviewer for pointing this out. For consistency with the reference calculations performed with sander, in this work we have used the sander interface of emle-engine, for which the bottleneck is the communication between sander and emle-server. Nevertheless, the MD performance is at least 2 orders of magnitude higher than that of sander+ORCA running on a single core. As suggested, we have performed additional benchmark QM/MM calculations with sander+QUICK. While it is indeed significantly more performant than the CPU-based ORCA code, it is still an order of magnitude slower than (non-optimized) ANI-2x(EMLE)/MM simulations. For a more realistic estimate of what kind of performance one can expect from ML/MM simulations with EMLE embedding, we also provide results obtained with the TorchScript based implementation of emle-engine coupled with OpenMM through the Sire framework. For the same system we obtained performances of 6 ns/day, 3 orders of magnitude better than sander+QUICK and 4 orders of magnitude better than sander+ORCA. A discussion of these points was added to the revised manuscript.

Minor Comments:
- *Figure 2: The resolution is too low.*
- *Page 4, Line 3: NMA is defined but never used again.*
- *Page 4, Line 6: The sentence "A more attractive…" is redundant with "A remedy for this problem…" on Page 3, Line 41.*
- *Page 4, Lines 26, 50, 55: QM, and MM, have not been defined previously.*
- *Page 4, Line 55: ML has not been defined previously (and maybe it should say MLP)*

- *Page 5, Line 17: In vacuo should be italicized.*
- *Page 7, Line 14: The basis set used to train ANI-2x is missing.*
- *Page 7, Line 17: In vacuo should be italicized.*
- *Page 7, Line 40: "The" Torchani package?*
- *Page 8, Line 3: Define La and Lb states for non-specialist readers; cutoff values for QM/MM and ML/MM are missing.*
- *Page 8, Line 31: Should it be "The solvated system"?*
- *Page 12, Line 41: There is a "." before "whereas"; consider revising to "display" and "but lack the one at...".*
- *Page 13, Line 22: PES has not been defined previously.*
- *Page 13, Line 34: Mention that 3-methylindole is a relatively rigid system when discussing PES consistency between ML/MM and QM/MM*

We thank the reviewer for their careful reading. All points have been addressed in the revised manuscript.

Reviewer 2

Recommendation: This paper may be publishable, but major revision is needed; I would like to be invited to review any future revision.

Comments:
In this work by Zinovjev and Curutchet, the authors present a comparison of excited state properties and spectral densities obtained from sampling configurations from either an MM force field trajectory or from a machine learned potential using the AN1-2x ML potential combined with an embedding scheme to account for solvent environment. The results show that energies and spectral densities agree well with those computed from a QM/MM trajectory, where the QM DFT method is the same wB97X functional used to train the AN1-2x ML potential. Overall, this seems like a very promising approach, and being able to reproduce the spectral density definitely showcases the abilities of the method. However, it is difficult to assess the comparison of the approach with the MM sampling, as there are no details given about the MM forcefield. Was it parametrized to use the same wB97X charges? Was it parametrized with other ab initio data (e.g. the Hessian)? Beyond this lapse in information, there are additional ways that the authors can improve the manuscript.

We are grateful for the positive evaluation of the manuscript and the constructive remarks quoted by the reviewer.

We apologize for the missing information on how the MM charges were parameterized. Our scope is to compare the behaviour of QM/MM and ML/MM with standard force fields used in biosimulations, although we understand better results can be obtained using more sophisticated MM parametrization strategies based on QM data. Thus, we adopted the Amber ff14SB force field, and 3MI was described adopting the bonded and Lennard-Jones parameters corresponding

to the side chain of Trp in ff14SB, whereas RESP charges where derived at the HF/6-31G(d) level of theory on B3LYP/cc-pVTZ optimized geometries. This is now clarified in the article.

In the revised version, following a request from reviewer 1, we have extended this study to a Trp embedded in the human serum albumin protein. Here again we used an Amber force field description for Trp, and our results indicate a very similar disagreement between MM-derived spectral densities and those derived from QM/MM and ML/MM, which agree very well.

1) Figure 1 would benefit from a more detailed caption, with a) b) c) labels explaining each component.

We agree with the reviewer, we have thus included a more detailed caption and labels for each panel explaining each step in our multiscale protocol to derive spectral densities.

2) In the paragraph beginning, "Here, we combine EMLE simulations...", I suggest modifying the sentence "We thus generated structures of 3MI..." to "We thus generated structures of 3MI solvated in water..."

Thanks for the remark. As now we also provide simulations in the HSA protein, we have changed the sentence to “We thus generated structures of 3MI solvated in water and embedded in the HSA protein at the ANI-2x/MM level of theory“.

3) It is unclear to me if there is any mixing of the La and Lb states during the trajectories. Can the authors add a plot to the SI with the La and Lb state energies as a function of time? Also, it would be good to give the specific details of how they identified each state, e.g. from alignment of the transition dipole with the ground state dipole?

This is indeed a delicate aspect that complicates analysis of the two excited states along the simulations, as there is significant mixing in some frames. In the new Fig S5 from the Supporting Information we now show representative examples of the L_a/L_b energies in trajectories obtained in the different environments, where one can see crossings in the ordering of the states during the simulations.

We have also clarified that the identification of the states was done based on the orientation of the transition dipoles, using as references the position vector from atom NE1 to CE3 to assign L_a, and the vector CG to CZ2 to assign L_b (atom names from Trp definition in Amber force fields). Overall, this assignment led to average values in our simulations deviating ~10-20 and ~30-45 degrees from the references for L_a and L_b, respectively, indicating that state mixing was small. We acknowledge however that the specific definition of the references can impact somewhat our results, as for some frames mixing leads to significant changes on the orientation of the transition dipole moments from the references.

4) Is the y-axis in Figure 2 a mean signed error? Similarly, is Table 1 showing average values?

We thank the reviewer for this remark. In Table 1 we show indeed average values, we have updated the caption to clarify this. In Figure 2, then, we report the errors between these average values compared to the QM/MM reference, not a mean signed error. We have also updated the caption of the Figure to clarify this.

5) In the spectral density figure, it seems that the gas phase spectral densities are much lower resolution. Were these from a long enough trajectory? I don't understand why they are much lower quality than the solvated results.

This observation is indeed interesting, as the MD trajectories in vacuum are of the same length as those in water solution (or the ones now reported in the HSA protein). One could expect that a shorter sampling is needed in vacuum compared to condensed phase, but the resulting spectral densities (SD) look less smooth, both in our TD-ωB97X results and those based on TD-B3LYP reported in the Supporting Information.

In order to shed light into this, we have computed the SDs in vacuum using the vertical gradient (VG) method, which is based on a DFT normal mode analysis of the ground state and TD-DFT calculations of the vertical gradient along those modes. Thus, these spectral densities are not affected by sampling, but one needs to apply a broadening to provide a realistic comparison with those derived from MD, here we used a Lorentzian lineshape with HWHM values of 15 and 35 cm^{-1}.

In the new Fig. S9 and S10 we compare the VG SDs (in vacuum) with those derived from MD in gas phase and in water, both at ωB97X and B3LYP levels of theory. Interestingly, the SDs obtained using both approaches in gas phase are in good agreement, once a broadening with a HWHM of 35 cm^{-1} is applied. In contrast, the water SDs derived from MD are in better agreement with the VG SDs derived adopting a HWHM of 15 cm^{-1}. We thus concluded that a larger broadening experienced by the vibrations in vacuum explain why they are less smooth than those obtained in solution, as they probably would require larger samplings to be well converged.

jz-2024-02949z.R2

Name: Peer Review Information for "Improved description of environment and vibronic effects with electrostatically embedded ML potentials"

Second Round of Reviewer Comments

Reviewer: 1

Comments to the Author

I greatly appreciate the significant effort the authors have put into revising their manuscript and carefully addressing each of the reviewers' comments in the revised version.

In my view, the article is now publishable in its current form, and I am confident it will be of great interest to researchers in this field.

Author's Response to Peer Review Comments:

Dear Editor

Please, find attached the manuscript, SI and TOC figure updated as requested.

Note that the references 14, 16 and 33 are identified by a code rather than pages, so I added the page ranges as 1-X.
